# Efficacy of neck-specific exercises with and without internet-based support on psychological factors in chronic whiplash-associated disorders: secondary analyses of a randomized controlled trial

**DOI:** 10.1093/pm/pnaf179

**Published:** 2025-12-23

**Authors:** Hedvig Zetterberg, Katja Boersma, Anneli Peolsson, Gunnel Peterson

**Affiliations:** Department of Psychology and Social Work, Mid Sweden University, Östersund, SE-831 25, Sweden; Center for Health and Medical Psychology (CHAMP), School of Behavioral, Social, and Legal Sciences, Örebro University, Örebro, SE-701 82, Sweden; Center for Health and Medical Psychology (CHAMP), School of Behavioral, Social, and Legal Sciences, Örebro University, Örebro, SE-701 82, Sweden; Department of Health Medicine and Caring Sciences, Unit of Physiotherapy, Linköping University, Linköping, SE-581 83, Sweden; Occupational and Environmental Medicine Centre, Department of Health Medicine and Caring Sciences, Unit of Clinical Medicine, Linköping University, Linköping, SE-581 85, Sweden; Department of Health Medicine and Caring Science, Endocrinology Centre, Linköping University hospital, Unit of Diagnostics and Specialist Medicine, Linköping University, Linköping, SE-581 85, Sweden; Department of Health Medicine and Caring Sciences, Unit of Physiotherapy, Linköping University, Linköping, SE-581 83, Sweden; Centre for Clinical Research Sörmland, Uppsala University, Eskilstuna, SE-631 88, Sweden

**Keywords:** whiplash-associated disorder, internet-based intervention, neck-specific exercise, physiotherapy, psychological outcomes, randomized controlled trial

## Abstract

**Objective:**

Chronic whiplash-associated disorders (WAD) can involve complex interactions between physical symptoms and psychological factors, and long-term effects of exercise programs are of interest. This study aimed to evaluate the effects of a predominantly internet-based neck-specific exercise program (NSEIT) compared with the same neck-specific exercises (NSE) performed at a physiotherapy clinic on psychological outcomes in chronic WAD, including cognitive-behavioral factors (pain catastrophizing, fear-avoidance beliefs, self-efficacy), emotional distress (symptoms of depression, anxiety), and cognitive functioning. A further objective was to assess whether psychological factors moderated treatment effects on the primary outcome, neck-specific disability.

**Methods:**

This secondary analysis was based on a randomized multicenter trial. Participants (*n* = 140) were randomized to NSEIT or NSE. Outcomes were assessed at baseline, 3 months, and 15 months. Psychological outcomes were analyzed with mixed-design analysis of variance. Moderation effects were tested by adding interaction terms (psychological factor×group) in linear regression models of neck-specific disability.

**Results:**

No significant group differences or group×time interactions were found for psychological outcomes. However, there were significant main effects of time for cognitive-behavioral factors and emotional distress in both groups (all *P* < .01), with small to intermediate effect sizes. Psychological factors did not moderate the treatment effects on neck-specific disability.

**Conclusion:**

The internet-based format (NSEIT), involving fewer clinical visits, did not differ from the more comprehensive physiotherapist-led program (NSE) with regard to psychological outcomes. Improvements in cognitive-behavioral factors and emotional distress occurred over time in both groups. The internet-based format might be suitable regardless of psychological characteristics at baseline.

**Clinical trial registration:**

www.ClinicalTrials.gov ID: NCT03022812 (December 20, 2016).

## Teaser

This study compared a predominantly internet-based neck-specific exercise program (NSEIT) with a physiotherapist-led version (NSE) in patients with chronic whiplash-associated disorders. Both groups showed similar improvements in cognitive-behavioral factors and emotional distress over 15 months, with no between-group differences. Psychological characteristics did not moderate treatment effects on neck disability. Findings suggest that NSEIT may be a suitable and accessible alternative to clinic-based treatment regardless of baseline psychological burden.

## Introduction

Whiplash-associated disorders (WAD) are common after motor vehicle collisions and are characterized by a cluster of symptoms, including neck pain, psychological distress, and reduced daily function.^1^^,^^2^ Although many individuals recover within a few months, about 50% develop persistent symptoms lasting >6 months, hereafter referred to as chronic WAD.^1^^,^^3^ Chronic WAD imposes a substantial individual and societal burden,^1^^,^^2^ and its management remains a clinical challenge.

The persistence of symptoms beyond the first months is more closely linked to high initial pain and disability and to post-injury psychological factors than to collision-related factors.^1^^,^^4^ In the integrated model of chronic WAD proposed by Walton and Elliott,^5^ the trauma is conceptualized as a trigger for a cascade of interacting physiological and psychological events—including altered neck-muscle function—which can lead to increased and maintained problems.^5^^,^^6^ Psychological risk factors for WAD chronicity include poor expectations of recovery, post-traumatic stress symptoms, and passive coping in the acute phase.^1^^,^^4^^,^^7^ Expectancies, pain catastrophizing, and fear of movement can further exacerbate problems,^4^^,^^7^ as described in the fear-avoidance model.^8^^,^^9^ Depression and anxiety are associated with, but not consistently prognostic for, developing chronic WAD,^1^^,^^7^ and a subgroup of patients with comorbid pain and depression can be identified.^10^ Consequently, psychological outcomes are important to monitor after WAD treatment.

Although psychological factors are evidently related to pain and disability in WAD, these factors have also been pointed out as potential moderators of treatment effect^11^^,^^12^—that is, whether the effectiveness of an intervention differs according to an individual’s psychological profile. If so, treatment could be stratified to risk profile to optimize resources and results.^13^^,^^14^ Moderation effects have been pointed out as a prioritized research area for commonly used interventions in WAD.^15^ Potentially, stratification could cover dose and delivery of treatment, in addition to content.

Exercise and patient education are currently the primary recommended interventions for chronic WAD,^15^^,^^16^ yet optimal delivery remains uncertain. Over a series of studies,^17–19^ a 3-month program of physiotherapy-led neck-specific exercises has been shown to be effective in chronic WAD grades II and III as compared with prescribed physical activity, across primary and secondary outcomes.^18–20^ However, twice-weekly visits to a physiotherapist clinic are resource intensive. A recent randomized controlled noninferiority trial therefore compared the same program to an internet-based version, with only 4 visits to a physiotherapy clinic.^21^ The programs were equally effective with regard to neck pain and neck-specific disability,^22^ which supports the conclusion that the internet-based program is sufficient to reach improvements.

The aim of the present study was to evaluate the effects of the neck-specific exercise program delivered via the internet with 4 visits to a physiotherapist (NSEIT) compared with the same exercises performed at a physiotherapy clinic (NSE) on secondary psychological outcomes in chronic WAD, with a 15-month follow-up. Psychological factors cover cognitive-behavioral factors (pain catastrophizing, fear-avoidance beliefs, self-efficacy), emotional distress (symptoms of depression, anxiety), and cognitive functioning. A secondary aim was to examine whether baseline psychological factors moderated effects on the primary outcome, neck-specific disability.

## Methods

### Design

This was a planned secondary analysis from a multicenter randomized controlled trial including 3 months’ and 15 months’ follow-up.^21^^,^^22^ The main trial, which tested the hypothesis that NSEIT would be noninferior to NSE, is described in detail elsewhere.^21^^,^^22^ Participants were recruited by advertising and were randomized to either a 3-month internet-based neck-specific exercise program with 4 visits to a physiotherapy clinic (NSEIT) or the same exercises and information given twice per week (optimum of 24 sessions) at a physiotherapy clinic (NSE). The study was approved by the regional ethics review board in Linköping, Sweden (Dnr 2016/135-31), and it was registered at ClinicalTrials.gov (protocol ID: NCT03022812; December 20, 2016). All participants provided written informed consent before study participation. Data were collected between April 6, 2017, and September 15, 2020.

### Participants

Recruitment was conducted through newspapers and media advertisements. Interested individuals completed an online questionnaire, followed by a telephone screening interview by research staff. Final eligibility was confirmed via physical examination by a trained physiotherapist, as described by Peterson et al.^22^

Inclusion criteria were persistent neck pain and disability after a whiplash injury at least 6 months, but less than 5 years, ago; WAD corresponding to grade II (neck pain, stiffness, tenderness, and clinical musculoskeletal signs) or III (grade II plus neurological signs); age 18–63 years; neck pain in the prior week ≥20 mm on the visual analog scale; neck disability >20% on Neck Disability Index; daily access to a computer, tablet, or smartphone and the internet; time to follow the treatment program; and neck pain, neck stiffness, or cervical radiculopathy within the first week after the injury. The upper age limit of 63 years was based on the intention that all participants would complete the 15-month follow-up before retirement (which at the time in Sweden was 65 years), because work ability was a secondary outcome of the main trial.

Exclusion criteria were any signs of head injury at the time of the whiplash injury; previous fractures or dislocation of the cervical column; known or suspected serious physical pathology; previous severe neck problems that resulted in sick leave for more than a month in the year before the current whiplash injury; cervical spine surgery; generalized or more dominant pain elsewhere in the body; other illness or injury that might prevent full participation; inability to understand and write in Swedish; diagnosed severe mental illness, such as psychosis, schizophrenia, or personality disorders; current alcohol and drug abuse; or participation in the earlier NSE study.^18^

### Sample size

Sample size and power were calculated on the basis of the primary outcome measure (Neck Disability Index) in the main trial.^22^ To detect a between-group noninferiority margin of 7% in Neck Disability Index with 1-sided α=0.025 and β=0.8, a total of 47 participants were needed in each group. The noninferiority margin was based on the recommendation for a minimal clinically important change in Neck Disability Index (7%).^23^ To account for dropouts, 70 participants were included in each of the 2 groups (140 in total).^22^

### Procedures

Included participants filled out a baseline questionnaire, after which the participant and the treating physiotherapist were informed of the participant’s group allocation, extensively described in Peterson and Peolsson (2023).^22^ A computer-based block randomization list stratified by sex was used for the randomization, which was conducted by an independent project member. Data collection at baseline and at 3- and 15-month follow-up was performed via online questionnaires on Linköping University’s Survey and Reports platform. The test leader was blinded to group allocation at baseline and at the 3- and 15-month follow-ups. The participant was blinded to group allocation when baseline data were collected.

### Interventions

The interventions, including photos of exercises, are detailed elsewhere.^22^ In the NSEIT group, participants received internet-based support in combination with 4 visits at the physiotherapy clinic over the 12-week intervention.^21^^,^^22^ The internet program provided participants with information, photos, and videos of all the exercises, as well as an exercise diary. The internet program also provided education on whiplash, pain, and pain coping strategies. Clinic sessions focused on exercise instruction, progression, and supervision of correct performance. Exercises were individually tailored. Participants in the NSE group received the same information and individualized exercises, but all were delivered in person by a physiotherapist at the clinic, with sessions scheduled twice per week for 12 weeks (optimum 24 sessions).^21^^,^^22^ In both groups, neurological pain during exercise was not permitted, and temporary muscle soreness was acceptable only if it did not worsen neck pain over time.^21^^,^^22^ Participants in both groups were encouraged to maintain neck-specific exercise after the 12-week program, as well as general physical activity 2 to 3 times per week, in line with existing recommendations.^24^ All treating physiotherapists completed 1-day theoretical and practical training provided by the project leaders and had access to support if needed. Physiotherapists followed a written protocol for the intervention and filled out session report forms after each session.

### Outcomes

Demographics and background characteristics were collected at baseline, including age, sex, level of education, occupational status, current neck pain intensity on a visual analog scale, and pain duration in months.

#### Psychological outcomes

Psychological outcomes were assessed via self-report questionnaires at baseline and at 3-month and 15-month follow-ups.

Pain catastrophizing was measured by the Pain Catastrophizing Scale (PCS).^25^ The PCS consists of 13 items assessing thoughts and feelings about pain (eg, “I worry all the time about whether the pain will end”). Items are rated from 0 (never) to 4 (all the time), with a total score range of 0–52. The PCS has shown good psychometric properties in pain populations.^26^ A cutoff score ≥30 indicates heightened levels of catastrophizing.^27^

Fear-avoidance beliefs were measured with the Fear-Avoidance Beliefs Questionnaire (FABQ),^28^ adapted for neck pain with good psychometric properties.^29^ The FABQ has 16 items covering fear and avoidance in relation to physical activity (eg, “Physical activity might harm my neck”) and work (eg, “I should not do my normal work with my present pain”). Items are rated from 0 (completely disagree) to 6 (completely agree). FABQ includes 2 subscales: physical activity (FABQ-PA; range 0–24) and work (FABQ-W; 0–42). Previously used cutoffs indicating elevated fear-avoidance are ≥15 for FABQ-PA and ≥28 for FABQ-W.^30^

Self-efficacy for daily activities despite neck pain was measured by the Self-Efficacy Scale (SES).^31^ The SES includes 20 items (eg, “drive a car,” “meet friends”) for which participants rate their self-efficacy to perform the activity from 0 (not at all) to 10 (very confident). Total scores range from 0 to 200, where higher scores indicate good self-efficacy.

Symptoms of depression and anxiety were assessed on the Hospital Anxiety and Depression Scale (HAD).^32^ The HAD includes 2 subscales for depression and anxiety, with 7 items each, for a total of 14 items. Items are rated from 0 (not at all) to 3 (very often). Each subscale has a total score range of 0–21, and the cutoff ≥11 has been used to indicate participants with depression and anxiety.^33^

Cognitive functioning was assessed with the Cognitive Failures Questionnaire (CFQ).^34^ The CFQ contains 25 items measuring lapses in attention, memory, and action. Participants rate occurrence of events from 0 (never) to 4 (very often). Total scores range from 0 to 100, where lower scores indicate good executive performance. The cutoff ≥43 has been reported to indicate worse performance.^34^

Trauma exposure and post-traumatic stress were also assessed. At baseline, participants were asked whether they had experienced a traumatic life event (eg, motor vehicle accident, other event). If they answered yes, they completed the specific trauma version of the Post-Traumatic Stress Disorder checklist (PCL-S).^35^ The PCL-S has a total score range of 17–85. The cutoff ≥44 has been reported to identify participants with post-traumatic stress disorder.^36^

#### Neck-specific disability outcome

Neck-specific disability was the primary outcome of the main trial^22^ and was included in the present study for moderation analyses. Neck-specific disability was assessed with the Neck Disability Index, which contains 10 items related to the impact of neck pain on daily activities. Items are rated from 0 (no activity limitation) to 5 (major activity limitation), and scores are transformed to a percentage scale ranging from 0% (no pain or disability) to 100% (highest score for pain and disability).^23^^,^^37^

Adverse events could be reported to the project leaders directly by participants, by the test leaders, or by the treating physiotherapists; for details, see Peterson and Peolsson (2023).^22^

### Statistical analyses

All statistical analyses were performed in IBM SPSS Statistics for Windows (Version 28.0; Armonk, NY; IBM Corp). Descriptive statistics were used to summarize baseline characteristics and outcome variables.

To address the first research question, a mixed-design analysis of variance (ANOVA) (2 treatment groups×3 time points) was used to evaluate changes in psychological outcomes over time. Analyses were based on available data (complete cases). The main outcomes were time (baseline / 3 months / 15 months)×group (NSEIT/NSE) interaction for each variable. In addition, main effects of group and time were analyzed, as well as simple within-group effects and between-group contrasts. Bonferroni correction was used for all pairwise contrasts (baseline vs 3 months, baseline vs 15 months, and between-group comparisons at each time point). Statistical significance was set at *P* < .05. Partial eta-squared (*η_p_^2^*) was reported as a measure of effect size; 0.01 indicates a small effect, 0.06 indicates an intermediate effect, and 0.14 indicates a large effect.^38^ The mixed-design ANOVA was used under the assumption that outcome data were multivariate and normally distributed and that any missing data were missing at random. This was supported by analysis of skewness (not greater than or less than 1), outliers (no extreme outliers according to Tukey’s test), and missing data (Little’s MCAR [missing completely at random] test nonsignificant for all variables). Mauchly’s test was used to test sphericity; if sphericity was violated (*P* < .05) the Greenhouse–Geisser corrected epsilon was used.

To answer the second question of whether psychological factors moderate effects on the outcome neck-specific disability, a series of multiple regression analyses was used. An interaction term was created for each psychological variable with the variable “group” (NSE/NSEIT, coded 0/1). The psychological variable, the variable “group,” and the interaction term were entered into the model as independent variables, with neck-related disability at 15 months’ follow-up as the dependent variable. Neck-related disability at baseline was included as a covariate. For the variable trauma (PCL-S), a dichotomized variable was used, coded 1 for the prevalence of traumatic life events.

## Results

**Figure 1.**
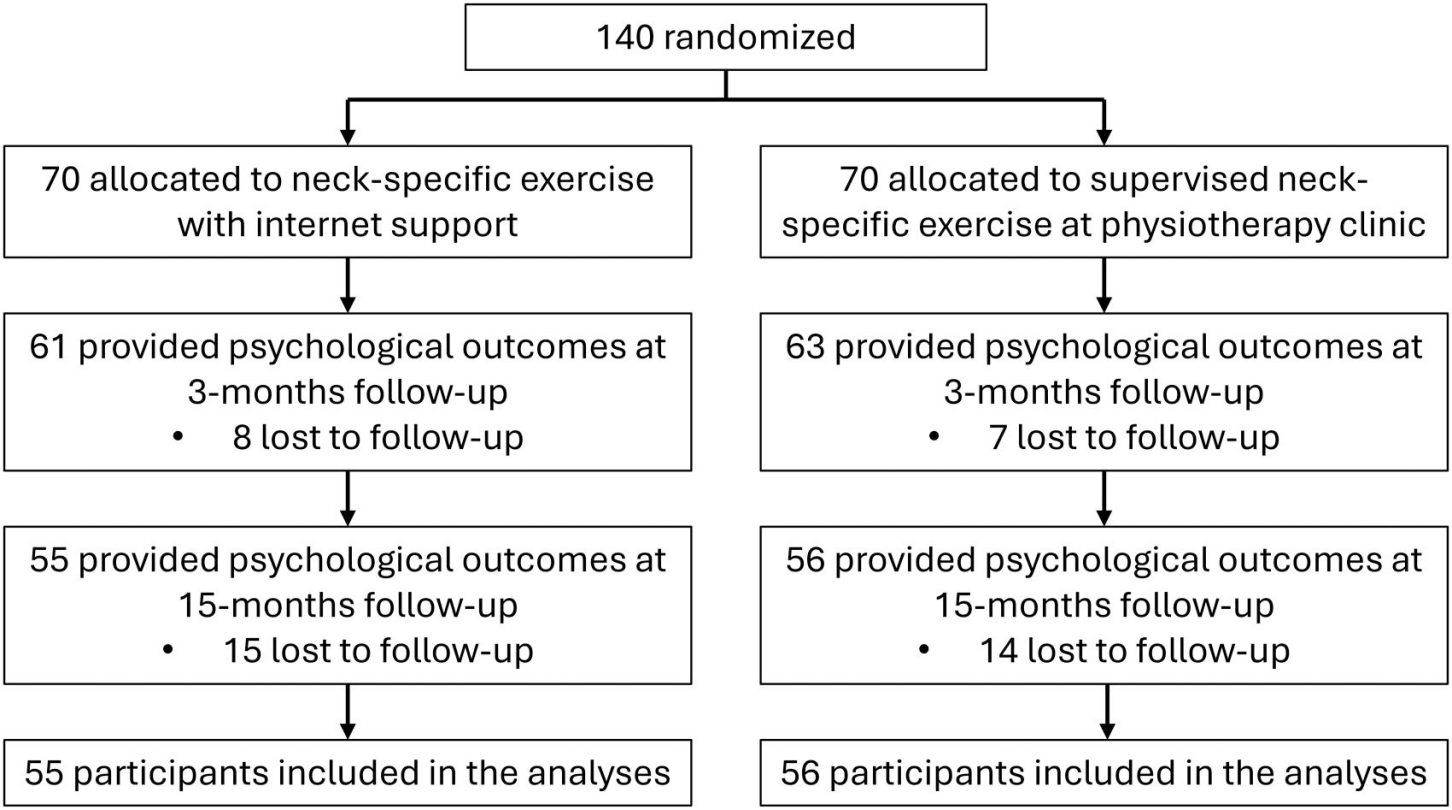
Flow chart of recruitment and available data.

A total of 140 participants were included and randomized in the main study, all of whom provided baseline data. With regard to the psychological outcome variables, 124 (89%) participants provided data at 3 months’ follow-up, and 111 (79%) participants provided data at 15 months’ follow-up; see Figure 1. There were no differences in baseline variables between the groups and no differences in attrition rate between the groups from baseline to 3 months and from baseline to 15 months. At both 3 and 15 months, nonresponders were younger than responders: At 3 months, the mean age was 35 years (SD 11) for nonresponders vs 41 years (SD 11) for responders, and at 15 months, the mean age was 35 years (SD 10) for nonresponders vs 42 years (SD 11) for responders. This difference was statistically significant (*t*_(138)_= −3.05; *P* = .003). Age was not correlated with change in any of the outcome variables. No adverse events were reported.

Participants’ characteristics are presented in Table 1. Most participants were women (79%), with a mean time since injury of approximately 2 years. About two thirds (64%) had WAD grade II and one third (36%) WAD grade III. Nearly all participants were employed or studying, and 17 (21%) were on part- or full-time sick leave.

**Table 1.** Participants’ characteristics at baseline, by treatment group.

	NSEIT (*n* = 70)	NSE (*n* = 70)
Age, mean (SD)	40.4 (11.6)	40.5 (11.4)
Months since injury, mean (SD)	27.4 (21.0)	25.2 (15.5)
Women, *n* (%)	55 (79%)	55 (79%)
Highest level of education, *n* (%)		
Elementary	1 (1%)	0 (0%)
High school	30 (43%)	39 (57%)
University	35 (50%)	27 (39%)
Other	4 (6%)	3 (4%)
Occupational status, *n* (%)		
Employed	63 (90%)	64 (91%)
Student	6 (9%)	6 (9%)
Unemployed / job seeking	1 (1%)	0 (0)
Sick leave, *n* (%)		
No sick leave	61 (87%)	62 (89%)
Sick leave, part time	6 (9%)	5 (7%)
Sick leave, full time	3 (4%)	3 4%)
WAD grade, *n* (%)		
Grade II	46 (66%)	43 (61%)
Grade III	24 (34%)	27 (39%)
Prevalence of traumatic life events, *n* (%)		
Car accident	32 (46%)	20 (29%)
Other or unknown	10 (14%)	17 (24%)
Post-traumatic symptoms (PCL-S, 17–85), mean (SD)	43.1 (14.4)	40.3 (13.3)
	(*n* = 41)	(*n* = 37)
Neck pain intensity now (VAS, 0–100 mm), mean (SD)	34.6 (21.4)	39.7 (22.4)
Neck Disability Index (0–100%), mean (SD)	39.4 (12.2)	36.6 (10.8)
Self-rated health (EQ-VAS, 0–100 mm), mean (SD)	57.7 (18.7)	58.6 (17.0)

*Abbreviations:* EQ = EuroQol; NSEIT = neck-specific exercises with internet support; NSE = neck-specific exercises at physiotherapy clinic; SD = standard deviation; VAS = visual analog scale; PCL-S = Post-Traumatic Stress Disorder Checklist—specific trauma version; WAD = whiplash-associated disorders.

### Effects on psychological outcomes

There was no interaction effect of time by treatment group on any of the outcome variables (all *P* *>* .05; Table 2), which indicates that patterns of change did not differ between treatment groups. However, as shown in Table 2, there were main effects of time on pain catastrophizing (*P* < .001), fear-avoidance beliefs (*P* < .01; *P* = .010), self-efficacy for daily activities (*P* = .001), and symptoms of depression and anxiety (*P* = .004; *P* < .001), which indicates that participants improved on these outcomes over time regardless of group assignment. Partial eta-squared ranged from 0.042 to 0.096 for these outcomes, which indicates small to intermediate effect sizes. Cognitive functioning showed no improvement over time (*P* = .490). Descriptive statistics for all outcome variables, as well as pairwise contrasts, are reported in Table 3. The pairwise contrasts revealed that the most consistent improvements were seen at the 15-month follow-up, specifically for pain catastrophizing, fear-avoidance beliefs related to physical activity, symptoms of anxiety, and self-efficacy for daily activities.

**Table 2.** Mixed-design ANOVA group (NSEIT/NSE)×time (baseline / 3 months / 15 months) results on cognitive-behavioral outcomes, including interaction and main effects.

Outcome and test	*F* statistic	*P* value	*η_p_^2^*
Pain catastrophizing (PCS)			
Group×time	*F* (2, 212) = 0.875	.418	0.008
Time	*F* (2, 212) = 9.077	**<.001**	0.079
Group	*F* (1, 106) = 0.028	.868	0.000
Fear-avoidance beliefs—physical activity (FABQ-PA)			
Group×time	*F* (2, 212) = 0.280	.756	0.003
Time	*F* (2, 212) = 10.358	**<.001**	0.089
Group	*F* (1, 106) = 0.018	.895	0.000
Fear avoidance beliefs—work (FABQ-W)			
Group×time	*F* (2, 214) = 0.102	.903	0.001
Time	*F* (2, 214) = 4.680	**.010**	0.042
Group	*F* (1, 107) = 0.175	.676	0.002
Symptoms of depression (HADS-D)			
Group×time	*F* (1.9, 203.7) = 1.098	.333	0.010
Time	*F* (1.9, 203.7) = 5.774	**.004**	0.051
Group	*F* (1, 108) = 1.322	.235	0.012
Symptoms of anxiety (HADS-A)			
Group×time	*F* (2, 216) = 0.706	.495	0.006
Time	*F* (2, 216) = 11.490	**<.001**	0.096
Group	*F* (1, 108) = 0.290	.591	0.003
Self-efficacy daily activities (SES)			
Group×time	*F* (2, 214) = 0.821	.441	0.008
Time	*F* (2, 214) = 6.728	**.001**	0.059
Group	*F* (1, 107) = 2.121	.148	0.019
Cognitive ability (CFQ)			
Group×time	*F* (2, 214) = 2.473	.087	0.023
Time	*F* (2, 214) = 0.717	.490	0.007
Group	*F* (1, 107) = 0.438	.510	0.004

*Abbreviations: η_p_*2 = partial eta-squared; CFQ = Cognitive Failures Questionnaire; FABQ = Fear-Avoidance Beliefs Questionnaire; HADS = Hospital Anxiety and Depression Scale; NDI = Neck Disability Index; NSEIT = neck-specific exercises with internet support; NSE = neck-specific exercises at physiotherapy clinic; PCS = Pain Catastrophizing Scale; SES = Self-Efficacy Scale. Significance (marked in bold) at *P* < .05.

**Table 3.** Outcomes and between-group effects for each treatment group at baseline and at 3- and 15-month follow-ups and within-group effects at 3- and 15-month follow-ups.

Outcome and group	Time points, mean (95% CI); *P* value			Within-group change, mean (95% CI); *P* value	
	Baseline	3 months	15 months	3 months—baseline	15 months—baseline
PCS					
NSEIT (*n* = 54)	18.1 (15.1 to 21.1)	14.6 (11.5 to 17.6)	13.8 (11.1 to 16.5)	−3.6 (−6.4 to −0.7); ***P* = .008**	−4.4 (−7.6 to −1.1); ***P* = .005**
NSE (*n* = 54)	16.6 (13.6 to 19.6)	15.4 (12.3 to 18.4)	13.6 (10.9 to 16.2)	−1.2 (−4.1 to 1.6); *P* = .871	−3.1 (−6.3 to 0.2); *P* = .074
Between-group effects	1.5 (−2.7 to 5.8); *P* = .480	−0.8 (−5.2 to 3.5); *P* = .711	0.2 (−3.6 to 4.0); *P* = .916		
FABQ-PA					
NSEIT (*n* = 52)	10.2 (8.4 to 11.9)	9.1 (7.5 to 10.7)	7.6 (6.1 to 9.1)	−1.1 (−2.9 to 0.8); *P* = .535	−2.6 (−4.5 to −0.6); ***P* = .005**
NSE (*n* = 56)	10.3 (8.6 to 12.0)	8.8 (7.3 to 10.4)	8.1 (6.6 to 9.6)	−1.5 (−3.3 to 0.4); *P* = .154	−2.2 (−4.1 to −0.3); ***P* = .015**
Between-group effects	0.1 (−2.6 to 2.3); *P* = .905	0.3 (−2.5 to 1.9); *P* = .802	−0.5 (−2.6 to 1.6); *P* = .635		
FABQ-W					
NSEIT (*n* = 54)	13.5 (10.7 to 16.2)	12.0 (9.3 to 14.8)	10.9 (8.2 to 13.5)	−1.4 (−4.1 to 1.2); *P* = .542	−2.6 (−5.3 to 0.0); *P* = .054
NSE (*n* = 55)	13.9 (11.2 to 16.6)	12.7 (10.0 to 15.4)	11.9 (9.3 to 14.6)	−1.2 (−3.8 to 1.4); *P* = .741	−2.0 (−4.6 to 0.7); *P* = .218
Between-group effects	−0.4 (−4.3 to 3.5); *P* = .827	−0.6 (−4.5 to 3.2); *P* = .744	−1.1 (−4.8 to 2.6); *P* = .562		
HADS-D					
NSEIT (*n* = 54)	6.1 (5.0 to 7.1)	5.2 (4.0 to 6.0)	5.5 (4.5 to 6.4)	−1.1 (−2.0 to 0.1); ***P* = .026**	−0.6 (−1.7 to 0.5); *P* = .558
NSE (*n* = 56)	5.4 (4.4 to 6.4)	4.7 (3.7 to 5.7)	4.3 (3.4 to 5.3)	−0.6 (−1.6 to 0.3); *P* = .275	−1.1 (−2.2 to 0.0); *P* = .058
Between-group effects	0.7 (−0.7 to 2.1); *P* = .430	0.3 (−1.1 to 1.7); *P* = .671	1.2 (−0.2 to 2.5); *P* = .094		
HADS-A					
NSEIT (*n* = 54)	6.9 (5.8 to 8.1)	5.5 (4.3 to 6.7)	5.7 (4.7 to 6.8)	−1.5 (−2.4 to −0.5); ***P* < .001**	−1.2 (−2.3 to −0.1); ***P* = .022**
NSE (*n* = 56)	6.4 (5.2 to 7.5)	5.5 (4.3 to 6.6)	5.1 (4.1 to 6.2)	−0.9 (−1.8 to 0.0); *P* = .068	−1.2 (−2.3 to −0.2); ***P* = .018**
Between-group effects	0.6 (−1.0 to 2.2); *P* = .468	0.0 (−1.7 to 1.7); *P* = .999	0.6 (−0.9 to 2.1); *P* = .436		
SES					
NSEIT (*n* = 54)	142.4 (123.2 to 152.6)	151.9 (141.9 to 161.9)	154.0 (144.1 to 163.9)	9.5 (−1.4 to 20.3); *P* = .108	11.6 (0.8 to 22.3); ***P* = .030**
NSE (*n* = 55)	153.7 (143.6 to 163.7)	156.1 (146.2 to 166.0)	165.2 (155.4 to 175.0)	2.5 (−8.3 to 13.2); *P* = 1.000	11.5 (0.9 to 22.2); ***P* = .028**
Between-group effects	−11.2 (−25.6 to 3.1); *P* = .122	−4.2 (−18.3 to 9.8); *P* = .551	−11.2 (−25.2 to 2.7); *P* = .113		
CFQ					
NSEIT (*n* = 53)	44.7 (40.0 to 49.4)	42.9 (38.0 to 47.8)	44.8 (40.0 to 49.6)	−1.7 (−6.2 to 2.7); *P* = 1.000	0.1 (−4.6 to 4.8); *P* = 1.000
NSE (*n* = 56)	42.9 (38.3 to 47.5)	43.8 (40.0 to 48.5)	39.7 (35.0 to 44.4)	0.8 (−3.5 to 5.1); *P* = 1.000	−3.2 (−7.8 to 1.4); *P* = .272
Between-group effects	1.8 (−4.8 to 8.3); *P* = .600	−0.8 (−7.6 to 6.0); *P* = .815	5.1 (−1.7 to 11.8); *P* = .138		

*Abbreviations:* CFQ = Cognitive Failures Questionnaire 0–100; FABQ-PA = Fear-Avoidance Beliefs Questionnaire Physical Activity 0–24; FABQ-W = Fear-Avoidance Beliefs Questionnaire Work 0–42; HADS-A = Hospital Anxiety and Depression Scale—Anxiety subscale 0–21; HADS-D = Hospital Anxiety and Depression Scale—Depression subscale 0–21; NSEIT = neck-specific exercises with internet support; NSE = neck-specific exercise at a physiotherapy clinic; PCS = Pain Catastrophizing Scale 0–52; SES = Self-Efficacy Scale 0–200.

All values are mean (95% confidence interval). Significance (marked in bold) at *P* < .05.

### Moderating role of psychological factors on the outcome neck-specific disability

As shown in Table 4, there were no interaction effects between the psychological factors and the treatment group on the primary outcome of neck-specific disability at 15 months’ follow-up after controlling for baseline levels of neck-specific disability. Psychological factors did not moderate treatment effects—that is, neither NSEIT nor NSE was more or less effective than the other depending on participants’ baseline psychological characteristics. Baseline levels of neck-specific disability were significant in all models, which indicates that more severe disability at baseline served as a general predictor of higher disability levels at 15-month follow-up (all *P* < .05; Table 4).

**Table 4.** Multiple regression analyses to the outcome Neck Disability Index at 15 months’ follow-up.

Variables	*β*	SE	Beta (standardized)	95% CI lower limit	95% CI upper limit	*P* value
PCS						
Group	0.408	4.191	0.014	−7.900	9.716	.923
PCS	0.088	0.140	0.067	−0.190	0.367	.530
NDI baseline	0.764	0.130	0.600	0.560	0.968	**<.001**
PCS×group	−0.066	0.203	−0.052	−0.468	0.336	.746
FABQ-PA						
Group	−1.472	4.325	−0.050	−10.047	7.102	.734
FABQ-PA	−0.139	0.237	−0.060	−0.609	0.331	.559
NDI baseline	0.800	0.108	0.626	0.587	1.014	**<.001**
FABQ-PA×group	0.062	0.366	0.027	−0.663	0.787	.865
FABQ-W						
Group	0.647	3.805	0.022	−6.986	8.190	.865
FABQ-W	−0.040	0.165	−0.027	−0.366	0.286	.807
NDI baseline	0.825	0.118	0.649	0.591	1.058	**<.001**
FABQ-W×group	−0.100	0.226	−0.064	−0.548	0.349	.661
HADS-D						
Group	−4.940	4.048	−0.167	−12.964	3.085	.225
HADS-D	−0.192	0.426	−0.050	−1.035	0.652	.654
NDI baseline	0.741	0.109	0.582	0.524	0.957	**<.001**
HADS-D×group	0.726	0.592	0.201	−0.447	1.899	.222
HADS-A						
Group	−0.312	4.162	−0.011	−8.561	7.937	.940
HADS-A	0.411	0.406	0.117	−0.394	1.217	.314
NDI baseline	0.748	0.100	0.587	0.549	0.946	**<.001**
HADS-A×group	−0.093	0.536	−0.029	−1.155	0.970	.863
SES						
Group	−4.702	9.925	−0.159	0.583	−0.079	.637
SES	0.031	0.055	0.078	−0.079	0.140	.583
NDI baseline	0.896	0.126	0.687	0.647	1.145	**<.001**
SES×group	0.028	0.064	0.145	−0.099	0.155	.666
CFQ						
Group	−4.552	6.098	−0.154	−16.640	7.536	.457
CFQ	−0.102	0.090	−0.121	−0.280	0.076	.260
NDI baseline	0.803	0.105	0.629	0.594	1.012	**<.001**
CFQ×group	0.090	0.131	0.154	−0.169	0.348	.494
Trauma						
Group	−0.151	3.423	−0.005	−6.937	6.634	.965
Trauma	0.812	3.148	0.027	−5.427	7.051	.797
NDI baseline	0.786	0.012	0.617	0.583	0.989	**<.001**
Trauma×group	−1.058	4.693	−0.032	−10.360	8.244	.822

*Notes:* Group 1 = NSEIT, 0 = NSE. Dependent variable = NDI at 15 months’ follow-up. Significance (marked in bold) at *P* < .05.

*Abbreviations:* CFQ = Cognitive Failures Questionnaire; FABQ-PA = Fear-Avoidance Beliefs Questionnaire Physical Activity; FABQ-W = Fear-Avoidance Beliefs Questionnaire Work; HADS-A = Hospital Anxiety and Depression Scale—Anxiety subscale; HADS-D = Hospital Anxiety and Depression Scale—Depression subscale; NDI = Neck Disability Index; NSEIT = neck-specific exercises with internet support; NSE = neck-specific exercises at physiotherapy clinic; PCS = Pain Catastrophizing Scale; SES = Self-Efficacy Scale.

## Discussion

This study presents secondary analyses from a recent randomized controlled trial comparing a program of internet-based neck-specific exercises and 4 visits to a physiotherapist (NSEIT) with the same program of exercises performed at a physiotherapy clinic (NSE).^21^^,^^22^ The present results focus on psychological outcomes. In summary, participants improved in cognitive-behavioral factors (pain catastrophizing, fear-avoidance beliefs, self-efficacy for daily activities) and emotional distress (symptoms of depression and anxiety). Cognitive functioning did not improve. Group allocation did not affect these patterns of change, which aligns with the main trial findings by Peterson and Peolsson^22^ showing that NSEIT was noninferior to NSE, with both groups demonstrating improvements in neck pain and neck-specific disability. The present findings support the conclusion that NSEIT with fewer clinical visits is sufficient to yield improvements in cognitive-behavioral factors and emotional distress similar to the improvements obtained with the more extensive clinic-based NSE.

Comparable findings were reported by Overmeer et al^20^ in a previous study comparing NSE with prescribed physical activity, in which improvements in pain catastrophizing, fear of movement, and anxiety were observed. The present study adds novel information on the efficacy of the internet-based format with fewer physiotherapist visits (NSEIT)—a relevant consideration based on the need for resource-effective interventions. Internet-based programs can also increase flexibility and accessibility for patients, thereby broadening the reach of comprehensive rehabilitation. The efficacy of digitalized physiotherapy interventions has been demonstrated across various patient populations.^39^^,^^40^ In the present study, the internet-based NSEIT was enhanced by clinical visits, providing an example of blended delivery of care.

In addition, the results showed that psychological factors did not moderate the effect of treatment on the primary outcome, neck-specific disability—that is, neither NSEIT nor NSE was more or less effective than the other according to participants’ psychological characteristics at baseline. This suggests that the digital format with fewer visits at a physiotherapy clinic (NSEIT) worked equally well as frequent clinical visits (NSE) for patients with elevated levels of pain catastrophizing, fear-avoidance beliefs, and symptoms of depression and anxiety or decreased levels of self-efficacy and cognitive functioning. Thus, an internet-based format with fewer face-to-face visits might be appropriate even for patients with a higher psychological burden. This has important implications for the clinical implementation of NSEIT. It should be emphasized that the core intervention content—neck-specific exercises and education—was identical in both treatment groups.

Among randomized controlled trials of exercise interventions in chronic WAD, evaluations of effects on psychological outcomes are scarce. However, 3 studies reported no or small improvements obtained by exercise interventions in the mental component score of the Short-Form 36 health survey.^12^^,^^41^^,^^42^ Among studies investigating psychological interventions (eg, exposure based) in subacute or chronic WAD, improvements in fear of movement, self-efficacy, and symptoms of depression and anxiety have been reported.^43^^,^^44^ The present study contributes to this literature by providing a comprehensive evaluation of psychological factors after 2 exercise programs, with follow-up extending to 15 months.

In the present study, effects on cognitive-behavioral factors and emotional distress were of small to intermediate magnitude. One likely reason for the modest effect sizes is the relatively low risk profile in the sample. For example, mean baseline values for pain catastrophizing, fear-avoidance beliefs, and symptoms of depression and anxiety were below the established cutoff values.^27^^,^^30^^,^^33^ Nevertheless, the improvements can be relevant, especially given that a cluster of factors seems to have improved.^22^ With regard to cognitive functioning, no group-level improvement was observed, which suggests that alternative intervention components might be needed to target cognitive symptoms. In the main trial, approximately 50% of the participants achieved clinically meaningful changes in pain and disability,^22^ which could partly explain the improvements in psychological outcomes reported here.

Although the mechanisms underlying treatment effects on WAD remain unclear, the improvements seen in cognitive-behavioral factors and emotional distress after NSEIT and NSE can be interpreted through a biopsychosocial framework.^5^ Neck-specific exercises target deep cervical muscle function, a factor potentially associated with pain and disability in chronic WAD.^6^ As suggested by Walton and Elliott,^5^ physical factors and psychological factors likely interact in multiple and bidirectional ways. A recent secondary analysis on the same sample demonstrated that improvements in psychological variables were associated with reduced neck pain and disability.^45^

In other musculoskeletal pain conditions, similar findings have been reported, in which exercise interventions are consistently linked to reductions in pain catastrophizing and improvements in self-efficacy.^46^^,^^47^ For example, Smeets et al^46^ found that reduced pain catastrophizing mediated the effects of both exercise programs and cognitive-behavioral therapy on pain and disability in patients with chronic low back pain. Physical exercises might involve exposure to new activities and increased function, which could challenge fear-avoidance beliefs and result in greater confidence in one’s own body.^46^ Importantly, both NSEIT and NSE included educational components targeting pain understanding and coping strategies,^22^ which could have contributed to improvements in cognitive-behavioral outcomes.^8^^,^^15^

Moreover, improvements in emotional distress can occur alongside changes in pain and disability, even when emotional distress is not explicitly targeted. For instance, Smith et al^48^ observed parallel changes in pain, disability, and emotional distress after cervical radiofrequency neurotomy. Burns et al^49^ further suggested that there might be reciprocal effects between process variables (eg, pain catastrophizing) and outcomes (eg, pain, depressive symptoms) in chronic pain, which could drive a process of mutual improvement. These findings support the notion that NSEIT and NSE might influence a cluster of interrelated symptoms in chronic WAD.^22^^,^^45^

Although no moderating effects of psychological factors on treatment format (NSEIT/NSE) were found, baseline neck-specific disability emerged as a consistent predictor of disability levels at 15-month follow-up. Specifically, patients with higher disability at baseline reported higher disability levels at follow-up, regardless of treatment format. Although these patients did show improvement, they remained more disabled than did those with lower initial levels of disability. This aligns with previous research.^50^ In a large cohort study of patients undergoing multimodal pain rehabilitation, the subgroup with the most severe initial profiles showed the greatest improvement.^51^ Similarly, in patients with chronic WAD, those with higher baseline levels of pain and disability experienced larger treatment effects from exercise compared with advice alone.^41^ These patterns suggest that patients with high psychosocial risk and disability could benefit from treatment, but combined exercise-based and psychological interventions^50^ or more extensive programs might be necessary for them to achieve sufficient recovery.

### Strengths and limitations

The present study provides secondary analyses from a randomized controlled multicenter trial with a 15-month follow-up, offering results on long-term effects on psychological outcomes. However, the sample size and power calculation were based on the primary outcome (neck-specific disability),^22^ which introduces a risk of underpowered secondary analyses into the present study. Nonetheless, findings were consistent with the main trial results^22^ and previous work on psychological outcomes.^20^ Moderation analyses require large samples to reliably detect effects, and results should be interpreted with caution. As previous studies from our research group had already evaluated NSE against a waiting list control group and a group receiving prescribed physical activity,^17–19^ the present randomized controlled trial did not include a third comparator arm but was narrowed to comparison between NSE in its original format and the version with internet-based support (NSEIT).

Finally, the sample in this study included WAD grades II and III, which strengthens the generalizability of the findings, especially because patients with WAD grade III are often excluded from clinical trials. However, baseline levels of psychological distress were generally low, which could limit generalizability to higher-risk populations. The exclusion criterion of generalized pain or more dominant pain elsewhere might have excluded those with greater psychological comorbidities and impacted the results. Low baseline levels on the outcomes might also have created a floor effect, limiting the chances of identifying group differences. Most participants in this study were women, which reflects the sex distribution commonly observed in populations with chronic WAD.^3^ However, the predominance of female participants could limit the generalizability of the findings to men, and sex- and gender-specific differences in treatment response warrant further investigation. In addition, participation required internet and computer or smartphone access, which might have excluded individuals with lower socioeconomic status in need of health care. However, internet and smartphone access are high in Sweden. Participants were recruited through advertisements, and treatments were delivered by physiotherapists in primary care. Future studies that include samples recruited through clinical settings would be of interest to further assess the effectiveness and implementation of the NSEIT in routine care.

### Conclusion

This study evaluated the efficacy of neck-specific exercise on secondary psychological outcomes in chronic WAD grades II and III, building on previous studies of whether a digital format with fewer visits to a physiotherapist (NSEIT) would be sufficient to reach effects similar to those previously demonstrated for a comprehensive physiotherapist-led program (NSE). There were no group differences between NSEIT and NSE in psychological outcomes, with small to intermediate improvements in cognitive-behavioral factors and emotional distress at 3-month and 15-month follow-up. Neck-specific exercise—whether delivered via internet-based support and fewer visits (NSEIT) or through regular visits to a physiotherapy clinic (NSE)—appears to be a promising treatment for patients with chronic WAD. Baseline psychological characteristics did not influence treatment response, which suggests that the internet-based format might be suitable even for patients with elevated psychological burden. However, patients with high initial neck-specific disability might require additional or extended treatment to achieve optimal outcomes. Further research into the interactions between exercise-based interventions and physical and psychosocial factors in chronic WAD is of interest.

## Data Availability

The data are available upon reasonable request.
